# A Wireless Sensor Network Deployment for Soil Moisture Monitoring in Precision Agriculture

**DOI:** 10.3390/s21217243

**Published:** 2021-10-30

**Authors:** Jaime Lloret, Sandra Sendra, Laura Garcia, Jose M. Jimenez

**Affiliations:** Instituto de Investigación Para la Gestión Integrada de Zonas Costeras (IGIC), Universitat Politècnica de València, Paraninf 1, 46730 Valencia, Spain; sansenco@upv.es (S.S.); laugarg2@teleco.upv.es (L.G.); jojiher@dcom.upv.es (J.M.J.)

**Keywords:** electromagnetic induction, soil moisture, precision agriculture, low cost, water management, Internet of Things (IoT), wireless sensor network

## Abstract

The use of precision agriculture is becoming more and more necessary to provide food for the world’s growing population, as well as to reduce environmental impact and enhance the usage of limited natural resources. One of the main drawbacks that hinder the use of precision agriculture is the cost of technological immersion in the sector. For farmers, it is necessary to provide low-cost and robust systems as well as reliability. Toward this end, this paper presents a wireless sensor network of low-cost sensor nodes for soil moisture that can help farmers optimize the irrigation processes in precision agriculture. Each wireless node is composed of four soil moisture sensors that are able to measure the moisture at different depths. Each sensor is composed of two coils wound onto a plastic pipe. The sensor operation is based on mutual induction between coils that allow monitoring the percentage of water content in the soil. Several prototypes with different features have been tested. The prototype that has offered better results has a winding ratio of 1:2 with 15 and 30 spires working at 93 kHz. We also have developed a specific communication protocol to improve the performance of the whole system. Finally, the wireless network was tested, in a real, cultivated plot of citrus trees, in terms of coverage and received signal strength indicator (RSSI) to check losses due to vegetation.

## 1. Introduction

Given the basic need to provide food to the world’s population, it is necessary to introduce technology to the agriculture sector to reduce the environmental impact caused by the crops and to increase the conservation of natural resources, among others [1]. Efficient Irrigation is one of the essential factors to increase the development of sustainable agriculture, especially in arid and semi-arid regions where there are the greatest limitations. Irrigation methods can be classified into three generic categories; these are (1) gravity irrigation, (2) sprinkler irrigation, and (3) drip irrigation. The gravity irrigation system is the oldest method and the least efficient for the conservation of natural resources. However, in order to determine the specific irrigation needs of crops, sensing devices must be deployed to obtain data such as soil moisture.

Precision agriculture is a concept that appeared in the USA in the 1980s. It is a management strategy that allows making decisions to improve farming productivity and to achieve more sustainable activity. It is based on the management of crops by observing, measuring, and acting against the variability of the many factors that affect them. Using Internet of Things (IoT) solutions, the soil where the crops are planted can be monitored to make decisions and perform more effective irrigation. These solutions may include not only the electronic devices deployed in the fields but also the use of vehicles such as drones to support the network [2] and to manage the use of pesticides on the crops [3,4]. However, in crop monitoring tasks, especially in those where fruit trees are grown, it is important to be able to control soil moisture levels accurately. For the correct progress of a fruit tree, it is necessary to ensure that the roots have the right levels of moisture. High humidity levels can facilitate the proliferation of fungi in the roots and leaves, thus affecting production. However, an extremely low soil moisture level can provoke the soil to crack. causing broken roots and the tree to die. This fact negatively affects the growth of plants and consequently their production.

One of the main drawbacks that hinder the use of precision agriculture is the cost of the sensors and the utilized technology. For farmers that want to use technology on a massive scale, it is necessary to provide low-cost systems to make easier deployments.

The available commercial sensors for soil monitoring use different methods to assess the water content of the soil. The most relevant existing methods for obtaining moisture values from the soil [5] are the gravimetric method, tensiometric method, neutronic method, gamma-ray attenuation method, dielectric method, Wenner or resistive method, and light method infrared [6]. Generally, when low-cost sensors are used to measure soil moisture, conductivity-based sensors are based on the use of two electrodes [1]. These types of sensors have two fundamental disadvantages, lack of reliability, and durability. On the one hand, depending on the type of soil and its salt content, the conductivity measurement can vary even when the amount of water in the soil is maintained. On the other hand, the electrodes must be in contact with the ground, and consequently, they can suffer rapid deterioration. Inductive sensors are also employed to measure soil moisture. However, they do not integrate the system into a sensor node to be able to read the parameters. 

The network design is an important aspect to consider as well. Usually, fields are located in remote areas. These areas may not have access to the internet infrastructure and the power grid. Therefore, PA systems should include a form of energy harvesting such as solar panels, and some characteristics of these networks should be considered when designing the deployment of sensing devices [7]. Wireless communications are a good solution because it eliminates the cost and hindrance of deploying cabled networks on extensive areas where machinery is utilized. However, the foliage of the crops affects the quality of the signal, resulting in reduced coverage between the devices. It is therefore necessary to determine the optimal deployment design for the area of interest according to the type of crop, and size of the field. Furthermore, the available protocols may not provide all the functionalities desired for a particular crop and the resources available for the area.

In this paper, we present a group-based wireless sensor network to efficiently irrigate cultivated lands. The network is composed of both actuators and sensor nodes that will collect data from the soil and will activate different irrigation systems as a function of the plot needs. Additionally, we design a new soil moisture sensor able to measure the amount of water content in the root ball of a tree. The design includes the sensor and the power circuit required to generate the bi-phase signals to power the coils. The paper presents the design of the operation algorithm and the message exchange for efficient use of water. Finally, the entire system is tested in a real environment to check the correct operation in terms of soil moisture measurements and network performance. 

The rest of the paper is structured as follows. Section 2 presents some previous and related works where soil moisture systems are developed. Section 3 presents an overall description of our proposed sensor as well as the features of the different coils used to develop our soil moisture sensor and the experimental tests performed with the coils. This section also includes the power circuit in charge of generating the required signals as well as the integration of both the sensor and the power circuit with an ESP32 module. Section 4 explains the network operation algorithm and message exchange between nodes. In Section 5, the tests performed in a real environment are shown. Section 6 explains the conclusion and future works.

## 2. Related Work

In this section, we summarize some previous works related to our proposal. The gap in current solutions for soil moisture monitoring is also identified. 

Authors such as Ojha et al. [8] present a study where they analyze the wireless sensor network (WSN) implementations for various agricultural applications. We will look at surveys such as the one presented by Garcia et al. [1], aimed at summarizing the current state of the art regarding smart irrigation systems and schemes for Internet of Things (IoT) irrigation monitoring. This survey includes the review of more than 100 scientific works. Other authors, such as Susha Lekshmi et al. [9], present a review of techniques employed for soil moisture measurement. The authors highlight the limitations of the techniques and the influence of soil parameters. Tumanski [10] describes the use of a coil to develop sensors. The work compares, summarizes, and analyzes coil design methods and frequency properties of the coil as well as the use of coil sensor applications such as magnetic antennas. Jawad et al. [11] describe applications of WSNs in agricultural research, and classify and compare wireless communication protocols, the taxonomy of energy efficiency, and energy harvesting techniques for WSNs used in agricultural monitoring systems. They also explore the challenges and limitations of WSNs in agriculture, highlighting energy reduction and agricultural management techniques for long-term monitoring. Hamami et al. [12] present a review of the application of WSNs in the field of irrigation. Mekonnen et al. [13] present a review of the application of different machine learning algorithms in the analysis of sensor data observed using WSNs in agriculture. In addition, they analyze a case study on a smart farm prototype, based on IoT data, as an integrated food, energy, and water (FEW) system. Nabi et al. [14] present a comparative study of different studies to provide a deeper insight into these implemented systems. They also present a study of apple disease prognostic systems, highlighting their key characteristics and drawbacks. The result of their study can be used to select appropriate technologies to build a WSN-based system, optimized for precision apple cultivation, which will help farmers avoid the ravages caused by disease outbreaks.

Kabashi et al. [15] present a framework to design WSNs for agricultural monitoring in developing regions, taking into account the particularities of said environments. They propose new solutions and research ideas for sensor network design, including zone-based joint topology control and power scheduling mechanism, multi-sink architecture with complementary routing associated with backlink/storage, and a task scheduling approach with parameter, energy, and environment recognition. Authors such as Kassim et al. [16] present WSNs as the best way to solve agricultural problems related to optimization of agricultural resources, decision support, and land monitoring in order to perform those functions in real time. They explain in detail the hardware architecture, network architecture, and software process control of the precision irrigation system. García et al. [7] study different WSN deployment configurations for a soil monitoring PA system, to identify the effects of the rural environment on the signal and to identify the key aspects to consider when designing a PA wireless network. The PA system is described, providing the architecture, the node design, and the algorithm that determines the irrigation requirements. The results of their testbed show high variability in densely vegetated areas. These results are analyzed to determine the theoretical maximum coverage for acceptable signal quality for each of the studied configurations. Furthermore, there are aspects of the rural environment and the deployment that affect the signal. Zervopoulos et al. [17] present the design and deployment of a WSN capable of facilitating the sensing aspects of smart and precision agriculture applications. They describe a simple synchronization scheme, which was installed in an olive grove, to provide time-correlated measurements using the receiving node’s clock as a reference. The obtained results indicate the general effectiveness of the system, although they appreciate a difference in the time correlation of the acquired measurements. Bayrakdar [18] investigated an intelligent insect pest detection technique with underground wireless sensor nodes for precision agriculture using a mathematical simulation model. To evaluate performance, he examined the received signal strength and path loss parameters. He observed the need for transmission of signals with different transmission powers for depth-based communication in wireless underground sensor networks.

Other authors study the application of WSNs to monitor specific crops. Khedo et al. [19] describe the implementation of the PotatoSense application, for precision agriculture with WSNs, to monitor a potato plantation field in Mauritius. They employ different energy efficiency algorithms, to ensure that the life of the system is prolonged. Additionally, they have developed a monitoring application to process the data obtained from the simulated WSN. Rasooli et al. [20] propose using WSNs and IoT to help increase wheat and saffron production in Afghanistan in the future. Using both techniques, they predict the control of the condition and growth of the crop as well as the ability to check soil, temperature, humidity, and other environmental parameters. 

Some authors propose the observation of parameters utilizing WSNs in greenhouses. Chaudhary et al. [21] propose and discuss the use of the programmable system on chip technology (PSoC) as part of the WSN to monitor and control various greenhouse parameters. Srbinovska et al. [22] propose a WSN architecture for vegetable greenhouses, in order to achieve scientific cultivation and reduce management costs from the aspect of environmental monitoring. They have designed a practical and low-cost greenhouse monitoring system based on wireless sensor network technology to monitor key environmental parameters such as temperature, humidity, and lighting.

There are authors also studying energy savings in WSNs used in monitoring agriculture. Hamouda et al. [23] study the problem of selecting the sampling interval, for precision agriculture using WSNs, due to the energy limitation that appears when deploying sensors in WSNs. They propose a Variable Sampling Interval Precision Agriculture (VSI-PA) system to measure and monitor agricultural parameters for appropriate agricultural activities, such as water irrigation. Compared to other fixed sampling interval schemes, the proposed VSI-PA system provides a significant improvement in energy consumption, while maintaining a small variation in soil moisture, regardless of soil temperature values. Qureshi et al. [24] propose Gateway Clustering Energy-Efficient Centroid (GCEEC)-based routing protocol, where a cluster head is selected from the centroid position and gateway nodes are selected from each cluster. The results obtained, after evaluating the proposed protocol in comparison to last-generation protocols, indicated a better performance of the proposed protocol, and provided a more feasible WSN-based monitoring for temperature, humidity, and lighting in the agricultural sector.

Table 1 summarizes different previous studies, carried out by other authors, regarding the use of WSNs in soil monitoring for agriculture.

Regarding the available sensors for soil monitoring, there are works, such as [25], that study farmed podzolic soils since these types of soils are under-represented in the relevant literature. In the study, the authors established the relationship between apparent electrical conductivity (ECa) and soil moisture content (SMC). The authors also evaluated the estimated SMC with ECa measurements obtained with two electromagnetic (EMI) induction sensors. The authors concluded that ECa measurements obtained through multi-coil or multi-frequency sensors had the potential to be successfully used for field-scale SMC mapping. Others, such as [26], designed and manufactured an integrated passive wireless sensor to monitor the moisture in the sand. The sensor was made of a printed spiral inductor embedded within the sand and it contained an inductive-capacitive (LC) resonant circuit. The authors measured the level of internal moisture by monitoring the resonance frequency using a sensing coil. Kizito et al. [27] presented a study where ECH20 sensors were used to measure soil moisture content, bulk electrical conductivity, and temperature for a range of soils, across a range of measurement frequencies between 5 and 150 MHz. The authors affirmed that the measurements carried out on soil were accurate enough to work at 70 MHz. Finally, Nor et al. [28] discussed the development of a low-cost sensor array based on planar electromagnetic sensors to determine the contamination levels of nitrate and sulfate in water sources. The authors proposed three types of sensors: parallel, star, and delta. According to their experiments, the star sensor array was the one with the highest sensitivity. 

After analyzing the exhibited works and many others not included in this paper, we can conclude that our work improves the existing systems. In either very few or no cases in the other works reviewed do the authors present complete or easily integrable systems in commercial nodes, such as Arduino or similar, and many of them use working frequencies that are too high (on the MHz scale). This fact makes it difficult to develop a simple and inexpensive signal generator circuit. Our proposal aims to take a step beyond the current state of the art, proposing a complete system, consisting of a sensor based on coils whose working frequency is around 93 kHz, and a power circuit that can be easily integrated into commercial modules for the development of a more complex wireless sensor network to monitor a large-scale crop.

## 3. Network Nodes Description

This section describes the proposed system and the different parts that comprise our proposed system. Additionally, it presents the features of the different coils used to develop our soil moisture sensor as well as the experimental tests performed to determine the best prototype.

### 3.1. Overall System Description

When trying to develop complete monitoring systems for precision agriculture, it is important to take into account different aspects. On the one hand, agriculture is an essential activity for the survival and development of society; this fact is evidenced by the amount of global- and regional-scale agricultural monitoring systems [29] to assess the crop growing conditions, crop status, and agro-climatic conditions that may have an impact on global production of any type of crop. Some examples are Group on Earth Observations Global Agricultural Monitoring Initiative (GEOGLAM) [30] or CropWatch [31], among others. 

On the other hand, it is necessary to know the kind of crop wanted to be developed in order to design adapted methods for monitoring activity. Considering the crop to monitor and the location of the plot, the network should use a specific wireless communication technology. Currently, it is possible to use cellular technologies by paying for subscriptions to a service or by using low-power technologies such as ZigBee, LoRa, LoRaWAN, Bluetooth BLE, or Sixfox, among others; most of these services do not require payment for using their communication network infrastructure [32]. However, the wireless technology par excellence for developing wireless sensors networks continues to be Wi-Fi. Although its energy consumption is still high, it allows transmitting any type of content without the bandwidth limitations that other technologies present. In addition, it is a widely studied standard so it is easy to develop new optimized protocols. Therefore, by making a good design of a power system based on renewable energies, it is possible to use Wi-Fi to develop a Wi-Fi-based agriculture monitoring network with very interesting properties. 

In the end, the completion of the design of the system led to precisely defining the type of parameter to be monitored since this fact will indicate the type of sampling, and analysis we should do. After that, the data interpretation and the scoring curves will help us to define the correct operation of our actuator network system. Lastly, the correct processing of collected data will help us to know the soil health and its characteristics for determining if these are optimal for our crop. 

**Figure 1:**
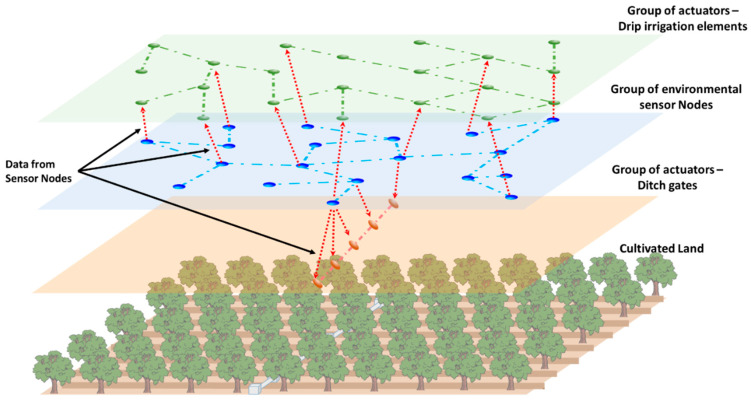
Proposed group-based network.

Therefore, considering these previous issues, we propose the development of a group-based wireless sensor network for soil moisture monitoring in precision agriculture. The network is composed of a set of nodes with different roles and functions. Some nodes are able to collect data from the environment, particularly data from soil moisture and other parameters required to ensure the correct progress of a tree (See Figure 1). The rest of the nodes have actuators to control the activity of ditch gates and drip irrigation elements. So, we will have 3 different sets of nodes that will communicate between them. Additionally, sensor nodes will provide data to the actuator nodes performing the required computation and decision making in the edge. Edge computing is recommended in scenarios where nodes present in the network are able to analyze the data and take decisions. Edge computing enables data produced by Internet of Things (IoT) devices to be processed closer to where it was created rather than being sent over long journeys to reach data centers and computing clouds. One of the fundamental advantages of this type of computing is that it allows analyzing important data in near real time [33]. In citrus groves, it is common to distribute them by forming rows of trees separated at a distance of approximately 6 m, being able to opt for a denser plantation, with a minimum separation of 4.5 m. The minimum depth that a citrus tree usually reaches is 45 cm. Considering these facts and taking into account that a field can have different extensions, different topologies of sensor nodes can be created. An important aspect is to ensure complete coverage between nodes to guarantee stable communication. A distributed ad-hoc network is optimal for this kind of scenario.

One of the main characteristic aspects of this proposal is its hierarchical structure by layers where each layer has a series of nodes that, if necessary, could change their role. That is, all sensor nodes and actuator nodes are wireless devices with the ability to act as a packet relay. In a hypothetical situation where a node falls, communications can be rerouted by other nodes of the same layer. If there is a fall of several nodes and one of them is isolated but active, it could use nodes of the upper or lower layer as an alternative way to carry out communications. However, these nodes would only forward the packet to nodes of the isolated node’s layer. 

To deal with the failure of a sensor or actuator node, it is convenient to establish an alarm system, based on keep-alive messages. It is a task periodically scheduled, once per day. It is possible to work with a large periodicity because the irrigation tasks of a field are not considered a critical task. If there is a node or several nodes not responding to these requests, the system will consider a node is down. 

Additionally, developing a low-cost system was required to measure the moisture in the soil depths. This system consists of four coil-based sensor elements equally distributed along 60 cm. The coils are connected to a processor module in charge of collecting the data and wirelessly share them with the rest of the nodes of its group. Finally, and considering the values of moistures collected by the sensor nodes, the actuator nodes will enable/disable the ditch gates or the drip irrigation.

When talking about moisture or soil humidity, we refer to the amount of water the soil contains. A gravimetric analysis method gives the relative comparison between the mass of dry soil and the mass of watered soil (which will always be higher). The moisture given in percentage is the result of dividing the difference between these two values by the mass of dry soil. If there is no difference, moisture will be 0%. In the opposite case, when the watered soil mass doubles the dry one, the moisture level will be 100%.

The development of our coil-based soil moisture sensor is based on the principle of electromagnetic induction of the coils and how it varies as a function of the type of core the coil has inside [34,35,36].

**Figure 2:**
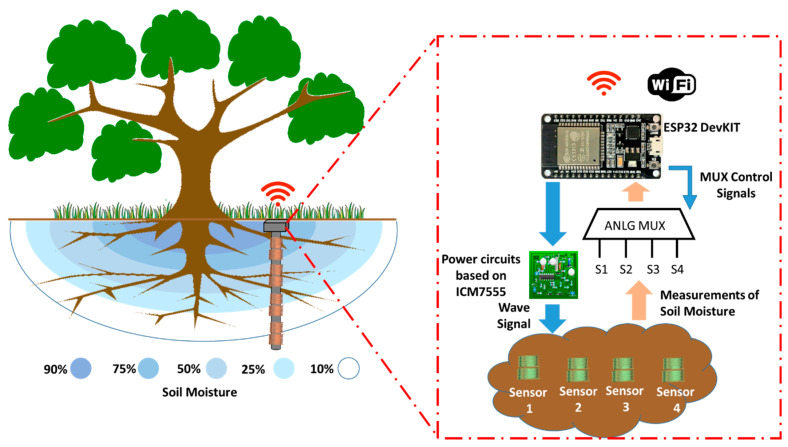
Diagram of proposed soil moisture sensor base on coils.

The soil moisture sensor is composed of two solenoid coils wound on the same PVC pipe support. Coil 1 receives the sinusoidal signal generated by the power circuit based on the integrated ICM7555. Coil 1 induces a current on Coil 2 which is largely affected by the content of the coil core since the magnetic field is affected by the type of soil and water content inside it. Finally, this current is measured, collected, and stored with an electronic module. In our case, a module ESP32 DevKIT [37] with an integrated Wi-Fi interface has been chosen. Figure 2 shows the diagram of the proposed soil moisture sensor.

Since this kind of module usually presents one or two analog inputs to collect data, we also propose the use of an analog multiplexor of four inputs which can be controlled by using two digital outputs. With this, our system will be able to take measurements from the four soil moisture sensors. 

### 3.2. Soil Moisture Sensor Based on Coils

As we mentioned before, it is possible to develop soil moisture sensors based on several principles and chemical processes. However, we want to use a method based on physical principles such as the variation of electromagnetic flow as a function of the nature of the coil core.

**Figure 3:**
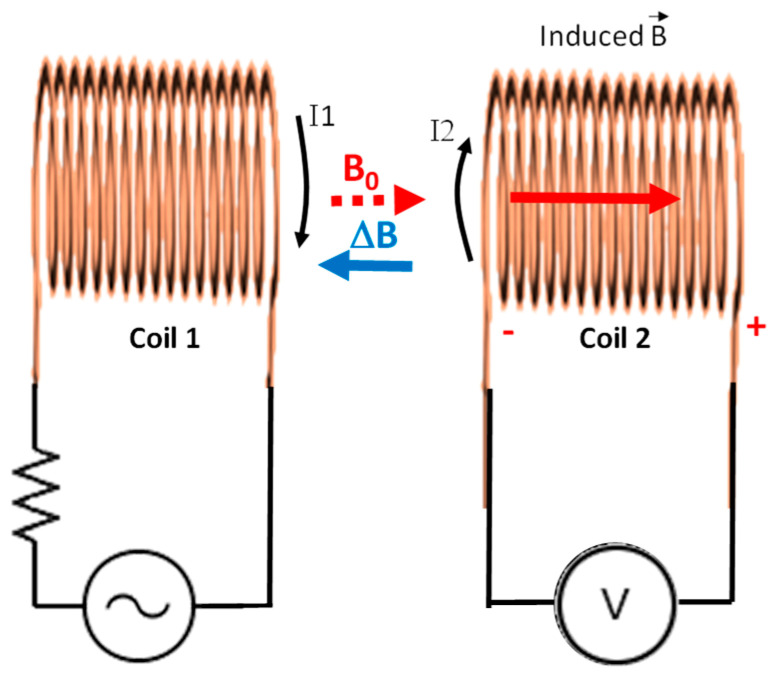
Principle of operation for our developed sensor.

In a coil distribution such as the one shown in Figure 3, coil 1 generates a magnetic field that affects coil 2. This effect is known as mutual inductance and refers to the electromotive force (EMF) in a coil due to the change of current in another coil attached. The induced EMF in a coil is described by Faraday’s law and its direction is always opposite to the change in the magnetic field produced in it by the coupled coil (Lenz’s law). The EMF in coil 1 (left) is due to its own inductance L.

The induced EMF in coil 2, generated by the changes of current I1, can be expressed as (see Equation (1)):

(1) $$emf_{2}=-N_{2}A\frac{\Delta B}{\Delta t}=-M\frac{\Delta I_{1}}{\Delta t}$$

where $N_{2}$ is the number of spires of coil 2, *M* the coefficient of mutual self-induction, *A* is the cross-sectional area of the coil, $\frac{\Delta B}{\Delta t}$ the variation of the magnetic field as a function of the time, and $\frac{\Delta I_{1}}{\Delta t}$ the variation of current in coil 1 as a function of time. Mutual inductance (*M*) can be defined as the ratio between the electromagnetic force (EMF) generated in coil 2, and the changes in current in coil 1 that causes that EMF. Likewise, *M* is highly affected by the characteristics of the medium that surrounds the coils, usually expressed by its magnetic permeability.

Since it is difficult to measure the value of the magnetic permeability of the earth core as a function of the moisture level, two theoretical approximations of the air core are introduced [38]. Based on Equation (2) (which presents the coil inductance), we can state Equation (3) where *l* is the length of the coil and *r* is the radius to the center of the coil of the innermost layer of the conductor while *R* is the radio for the outermost layer.

(2) $$L=\frac{\Phi N}{I}=\frac{\mu N^{2}A}{l}=\frac{\mu_{0}\mu_{r}N^{2}\pi r^{2}}{l}\;\left(H\right)$$

(3) $$L_{layer}=\frac{N^{2}r^{2}}{2.54\left(9r+10l\right)}\;\left(\mu H\right)$$

where *L* is the inductance of our coil (in H), Φ is the magnetic flow (in Wb), *N* represents the number of turns (dimensionless), *l* is the length of the coil (in m), *r* expresses the radius of the inner coil’s layer (in m), *R* is the radius of the outer coil’s layer (in m), *A* is the area of the coil’s surface (in m^2^), *µ*_0_ is the magnetic permeability (free space) (H/m) and finally, *µ_r_* is the relative magnetic permeability (medium) (dimensionless).

This approximation allows estimating the components of the circuit for an air core, which would be similar to those obtained with a large amount of pure water; so this value will vary depending on the type of soil, its composition, and the level of soil moisture presented by the soil that contains coil. The resonance peak of our coils can be calculated by Equation (4).

(4) $$f_{r}=\frac{1}{2\pi }\sqrt{\frac{1}{LC_{d}}-\frac{R_{S}^{2}}{L^{2}}}\approx \frac{1}{2\pi \sqrt{LC_{d}}}\;\left(\mathrm{Hz}\right)$$

where *f_r_* is resonance frequency (in Hz), *C_d_* is coil’s parasite capacity (in F), *L* is the coil’s inductance (in H) and, *R_s_* is the coil’s resistance (in Ω).

We should take into account that the primary coil and secondary coil will have different resonance frequencies because the secondary coil has a different number of coils. However, our sensor only intends to detect changes in the induced current due to the presence of a changeable medium and, finally, we want to relate this value of current with the amount of water content in the soil.

Equations from (1) to (3) are theoretical approaches to explain how important it is to know the relationship between the physical and electrical characteristics of the coil. Equations (1)–(3) explain how the coil inductance, and hence mutual inductance, depend on its geometry (length, radius, and the number of turns) for single-layer coils. Equation (4) helps us to design a resonant circuit to obtain the maximum power transfer. It is highly important to consider the appearance of a possible parasite capacity due to coil geometry and working frequency.

We previously performed several tests with different combinations of coils, varying the number of spires, the ratio of spires between coils, and the diameter [39]. In these previous works, we performed many experiments with combinations of spires and the best results were determined for a ratio of 1:2 with a medium value of spires and larger diameter. For a fixed diameter, if we reduced the number of spires, the working frequency increased for a fixed number of spires; if we increased the coil diameter, the working frequency decreased. Additionally, developing a simple and cheap electronic system to generate the signals was required. For such a system, it is highly recommended to have a sensor that requires the lowest working frequency. Therefore, we chose to set some parameters such as the type of copper and number of spires, we only varied the diameter of coils. 

**Figure 4:**
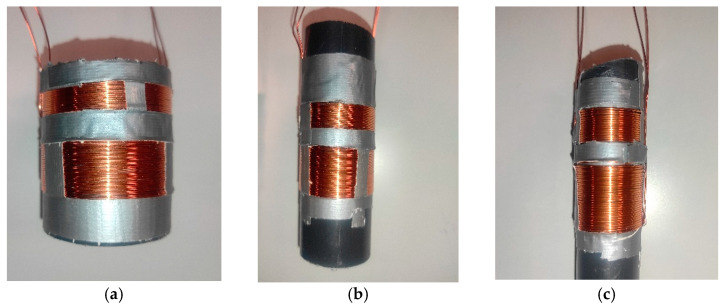
Coils used in our developed sensor: (**a**) P1, coil of 50 mm;(**b**) P2, coil of 32 mm; (**c**) P3, coil of 20 mm.

In developing our coils, 0.6 mm enameled copper wire was used. The process entails winding copper wire along a cylinder, forming two solenoids. The distance between the primary coil and the secondary coil is five mm. Figure 4 shows the developed coils with a single layer of spires. Table 2 shows the physical features of each prototype.

The procedure to perform these tests with the coils consists of introducing each model into a container filled with dry and compacted soil to observe the behavior of the output voltage as a function of the amount of water. Therefore, for the same moisture level, a frequency sweep will be carried out to find the frequency that shows a peak in the induced voltage. This value will be the sought-out resonance frequency. After that, the linearity of each model will be analyzed. For each test, 4000 g of soil is used, with increments of 250 mL of water, for each moisture level up to 1000 mL of water. When water is added to the soil, the sample is reposed for an hour to obtain a homogeneous sample inside the coil. Specifically, five levels of content of water in soil will be measured: 0%, 6.25%, 15.5%, 18.75%, and 25% (see Table 3). For this type of soil, 25% of the content of water in soil implies a land completely flooded. Measurements have been taken at 25 °C.

In order to determine in which type of soil our sensor can be used, we endeavored to determine which one presents the biggest linearity. The idea of this concept is that an increase in the percentage of moisture is equivalent to an increase in output voltage without instabilities.

### 3.3. Experimental Results with the Developed Coils

This subsection presents the test performed to determine the most suitable prototype selected to develop the system, followed by the testing of the selected coil with different types of soils and different levels of moisture. 

Considering the types of soil, we can conclude that soils are usually made up of different proportions of sand, silt, and clay. Each of them has morphological characteristics:

- Sandy soils, coarse texture (sand and clay sand).
- Silty soils, moderately coarse texture (sandy clay and fine sandy clay), medium texture (very fine sandy clay, silt, silt loam, and sediments), and moderately fine texture (clay silt, clay sand loam, and sand loam soil silty).
- Clay soils, fine texture (sandy clay, silty clay, and clay).

In order to perform our tests, we have selected three different types of soils, i.e., sand from the beach, soil from cultivated land, and commercial universal substrate. 

The sand on the beaches is formed by sediments from rocks and other marine debris such as shells, corals, animals, algae, and even sand that travels through the rivers until flowing into the sea. Due to the erosion of water and wind, due to rain and waves, or temperature differences, the grain size of the sand tends to be reduced.

The soil from cultivated land is usually made up of an organic fraction, organic matter more or less degraded into humus and humic and fulvic acids. These elements provide the fertile part of the earth. The rest of the soil is considered as physical support. Some farmlands have a high degree of clay which also intervenes in ion exchange and water retention, facilitating the release of fertile elements according to the needs of the plants.

The raw materials used in the manufacture of a commercial universal substrate are usually blonde peat from sphagnum moss, coconut fiber, compost, perlite, organic fertilizer, mineral fertilizer, algae extract, etc. In addition, this type of soil usually contains a high level of aeration.

Performing the measurements entails three identically constructed sensors simultaneously placed in three samples of each soil. The results shown in our graphs are the average value of the three measurements collected, which in all cases were identical. 

**Figure 5:**
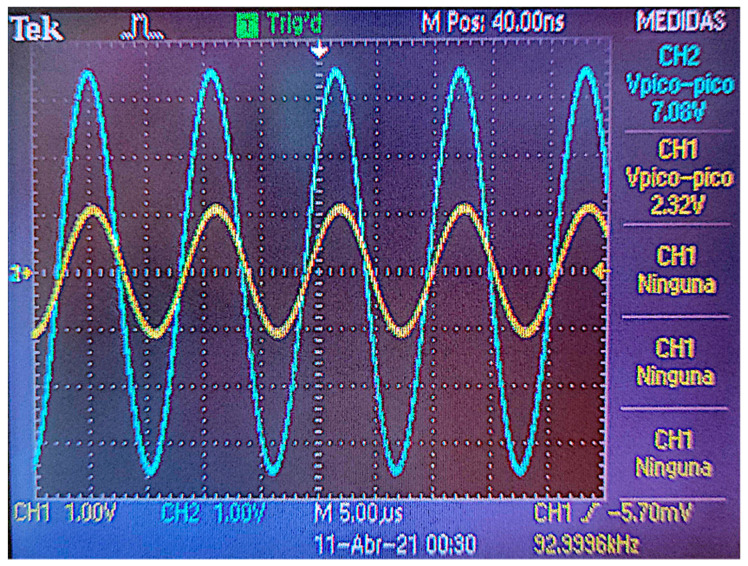
Example of generated and obtained signals.

In order to perform the test, the primary coil is powered by using a wave of 7 Vpp with positive and negative values. For example, it is possible to use sine or square waves, such as the one shown in Figure 5. In Figure 5, we can see, in blue, the signal used to power the primary coil while the result of the induced current is shown in yellow. 

**Figure 6:**
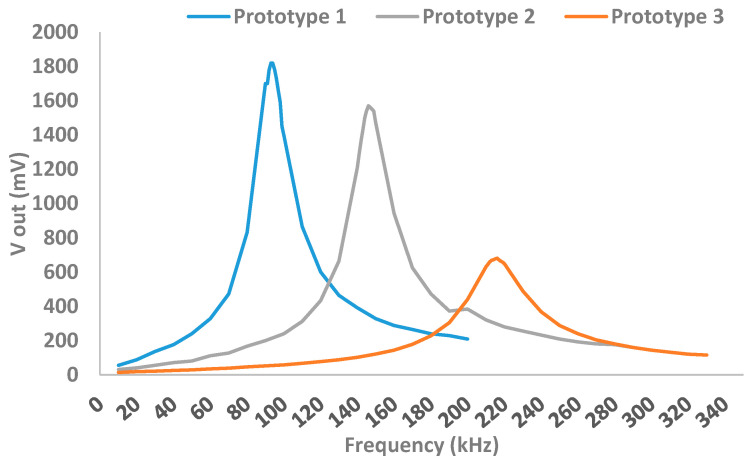
Resonance frequency obtained for each prototype.

Figure 6 shows the preliminary results obtained with the 3 coils. Figure 6 shows the value of the resonance frequency and the maximum voltage value.

Table 4 shows the resonance frequency values (in kHz) of the developed prototypes and the maximum voltage value (in mV) obtained in the induced coil.

**Figure 7:**
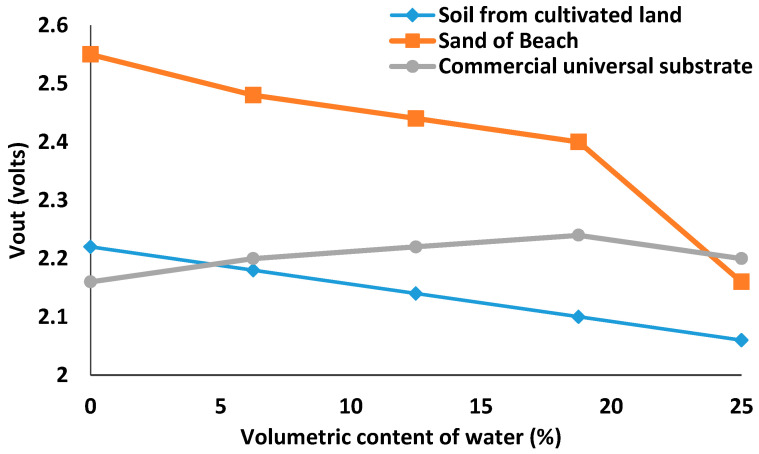
Behavior for prototype 1 in several types of soils.

After analyzing the results obtained in Figure 7, we can conclude that the prototype that gives the best results is Prototype 1, with a working frequency of 93 kHz and a maximum output voltage of 1.82 V.

Once the most suitable sensor for further development has been determined, this sensor is tested on different types of soil to determine its versatility. In all cases, we will look for the maximum linearity in the sensor response.

As we can see in the previous figure, the selected model has a linear behavior for all three cases up to a humidity degree of 18.75%, i.e., a total volume of water of 750 mL for 4000 g of sand.

Another important aspect to highlight is that the behavior of the sensor for a universal substrate is inverse to the behavior shown in the case of beach sand or cultivated soil. This aspect should be considered when the results are processed in a real environment.

### 3.4. Power Circuit Design

**Figure 8:**
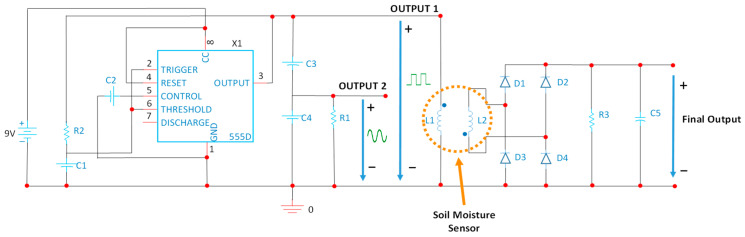
Enhanced power circuit schematic.

**Figure 9:**
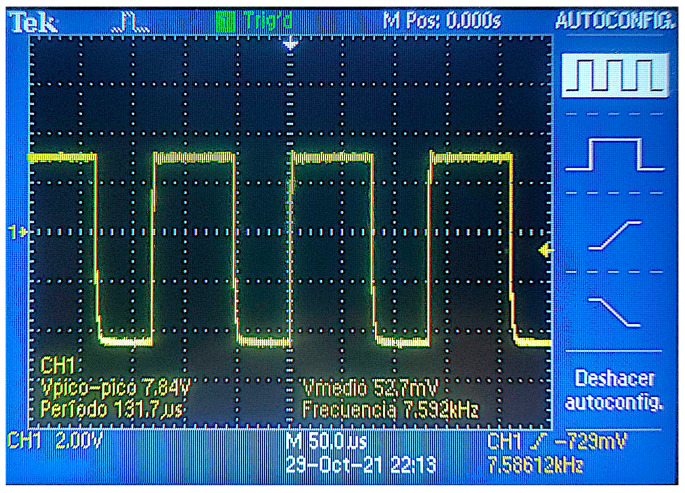
Example of signal obtained in Output 1.

Locating the resonance frequencies for the selected prototype of coil systems requires a power supply and excitation circuit able to generate an alternating signal. To do this, a 555 series oscillator integrated circuit [40] will be used. A series of components will be used to obtain the desired output signal with a resonance frequency of 93 kHz. Our circuit is based on the ICM7555 [41]. According to the manufacturers’ specifications, this integrated circuit can generate signals up to 3 MHz. Figure 8 shows the schema of our entire circuit. This kind of integrated circuit has been conceived to be customized regarding the duty cycle of signal and the required frequency. In this case, we can use R2 and C1 to change the working frequency while C3, C4, and R1 are used to control the ripple signal and its form. Modifying C4 and R1, it is possible to obtain both a sine wave and square signal, such as the one shown in Figure 9.

To directly read a value of voltage proportional to the amount of water content in the soil, we include a Graetz bridge or double wave rectifier bridge followed by an RC filter that has been connected to the terminals of the secondary coil. 

### 3.5. Comparision and Discussion with Existing Published Systems

We have compared our sensor model with existing and commercial soil moisture sensors. Table 5 shows this analysis. It is important to consider that this table only contains the price of the sensor, with exception of references [42,43,44,45] which include an electronic module. In the rest of the cases, a microprocessor module must be included similar to the one used in this paper that can cost approximately $10–$15.

It is evident that our proposed sensor, based on 2 coils, is one of the models that presents the lowest prices. The price includes the pipe and wire, because it can be added to any electronic platform to gather the data. 

When this type of system is designed and developed, it is extremely important to consider practical implementation problems and challenges. 

One of the main problems in the outdoors is how to protect the electronics from adverse conditions. A waterproof protection is highly recommended to protect the different devices since the places where the devices are deployed can be highly changeable. Additionally, the time during which the sensor nodes should work and the exposure to environmental temperature and humidity can cause some variations in the measurements. This issue should be controlled since a wrong reading would cause anomalous values and consequently wrong behavior. To shore up this problem, it is possible to use artificial intelligence and redundancy mechanisms. 

Furthermore, there is another important issue regarding manufacturing techniques of certain sensors and probes. In several cases, they are manufactured with copper. An improvement in the system implementation could be the replacement of these sensors for ones protected with the process of gold plating which help to combat the corrosion of probes. 

Coverage estimations do not usually match practical experimentation because the emulation of environmental conditions is difficult. For this reason, we highly recommend performing practical experiments and test benching, as presented in this paper.

## 4. Network Protocol Design and System Procedure

This section presents the network protocol used in our topology. In addition, it also presents the algorithm designed to collect data from sensors and control the different actuators as well as the messages exchanged between devices and the algorithm designed for the system procedure.

**Figure 10:**
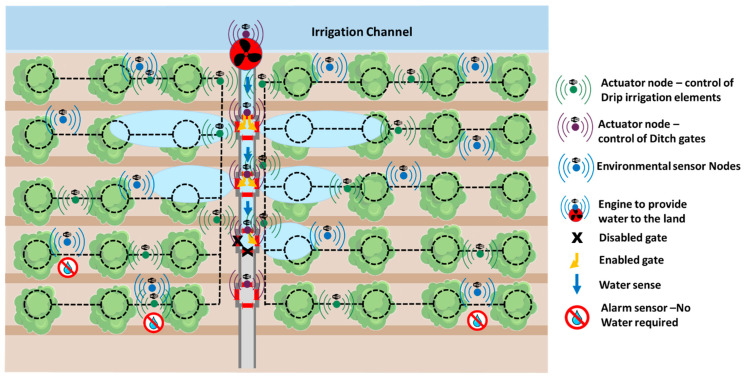
Diagram of our entire network in the plot.

As we presented before, our network is composed of three different types of nodes, which can be classified as sensor nodes and actuator nodes. Additionally, we consider an additional node that is placed in the engine to provide water to the plot. This node will be in charge of starting the process of monitoring the entire network. Sensor nodes collect data from the soil and provide the required warning alarms to the actuators for enabling or disabling the irrigation systems. Figure 10 shows the diagram of our entire network deployed in the plot.

In order to obtain high performance, we have developed a specific network protocol. This section presents the message exchange between devices, the fields of the messages exchanged between devices, and the algorithm designed for the system procedure.

The designed network is a distributed network made of sensor nodes (each one has one or several physical moisture sensors), and one or several actuators which activate the engine, for the drip irrigation system and/or the ditch gates, depending on the case.

Then the number of nodes (see Equation (5)) of the whole system (*N*) is:

(5) $$N=n_{s}+n_{a}$$

where *n_s_* is the number of sensor nodes and *n_a_* is the number of actuator nodes.

Our network will use Ad Hoc On-Demand Distance Vector Routing (AODV) since it is one of the ad hoc routing protocols that presents the best performance [53].

### 4.1. Algorithm of the System

**Figure 11:**
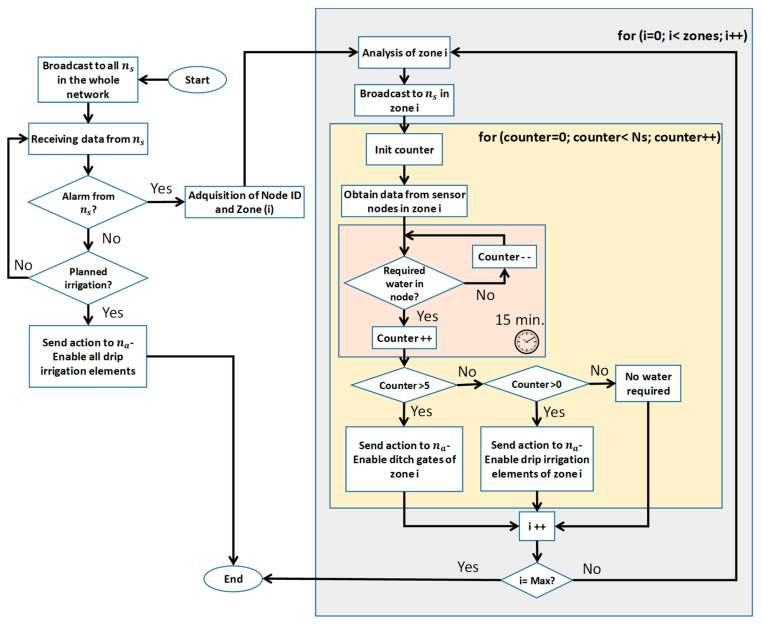
Operation algorithm.

In order to determine when the irrigation process should be carried out, we need to collect the data from the different $n_{s}$ which are placed and identified by zones (i). For each zone, we defined the maximum number of nodes comprised in the zone as counter. Carrying out this automation process of irrigation requires the design of an operation algorithm. Figure 11 shows the operation algorithm of our soil moisture monitoring system.

As commonly done in agriculture, there are periodic planned irrigations that should be performed. In this case, the system of drip irrigation elements is enabled and it covers the entire extension of trees. If an alarm is registered from a sensor, the system will request the data from all nodes of that zone. If the number of nodes that register the need for water is higher than 5, the system will enable the ditch gate of this zone. Even if only some sensors warn about the need for water, the system will enable the drip irrigation elements of this zone. The rest of the zones in the plot will be analyzed to check if it is required to proceed with irrigation. The different orders will be sent to the sink node by the nearest node of that area to the sink node which will be in charge of enabling/disabling the irrigations systems. 

Finally, if the plot does not require any action, the system will remain in idle mode waiting for new information. 

### 4.2. Message Flow between Nodes

**Figure 12:**
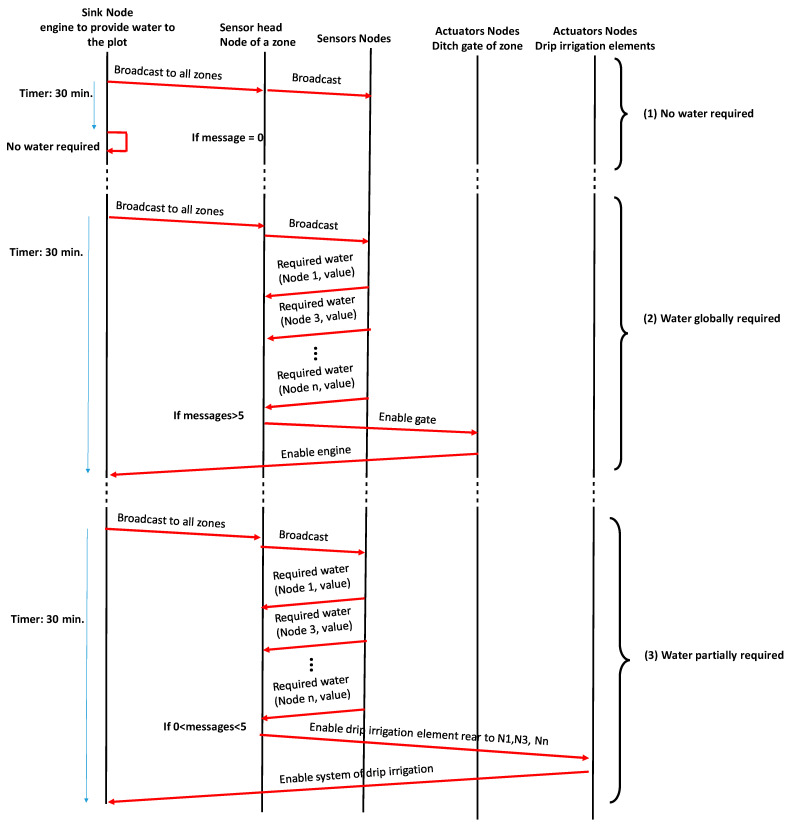
Message exchange between nodes.

Finally, in order to send the required actions to the correct actuator nodes, it is important to design the message exchange between nodes. In this sense, we should consider three different situations (see Figure 12). Firstly, the most frequent situation is the one in which the plot does not require any type of irrigation. In this case, if the sensor nodes do not send any message in the next 30 min, the system will consider that no irrigation is required (1). 

The second situation (2) is when there is a global need for water in a zone of the plot. In this case, the sensor head node will wait 30 min for messages from sensor nodes. If more than 5 messages are received, the system will consider that global irrigation for this zone is required. Then, the sensor head node will send a message to the actuator node in charge of enabling the gates. After that, this node will inform the sink node to enable the engine to provide water to the ditch.

The third situation (3) will be done when there is a partial need for water in a zone of the plot. In this case, the sensor head node will wait 30 min for messages from sensor nodes. If less than 5 messages are received, the system will consider that partial irrigation for this zone is required. Then, the sensor head node will send a message to the actuator node in charge of enabling the drip irrigation element of the affected trees. After that, this actuator node will signal the sink node to enable the system of drip irrigation.

To make easier the process of forwarding messages from sensor nodes to actuator nodes or sink nodes, it is possible to use any node present in the network. In this sense, a node can receive several packets but if it is not the destination of this message, the node will relay the message without processing it. When the sensor nodes of a zone are communicating, an intragroup routing protocol will be utilized. When the message exchange is performed between sensor nodes and actuator nodes or between actuator nodes for ditch gates and actuator nodes for drip irrigation elements, these nodes will use an intergroup routing protocol [54].

## 5. Experimental Results in a Practical Deployment

**Figure 13:**
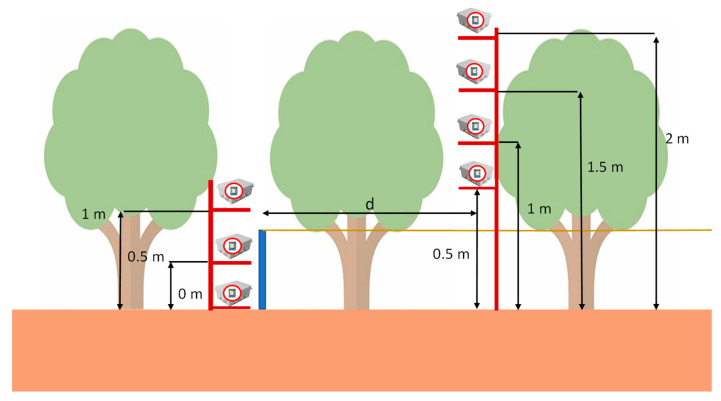
Testbed.

In this section, the results obtained in the deployments on orange groves are presented. In order to perform the test, we have used several ESP32 DevKit nodes placed at different heights. This will allow us to study the coverage of the nodes at different heights, so it will be kept as a recommendation for practical deployments. The different deployment strategies that were tested are presented in Figure 13. As can be seen, different configurations of emitter height and receiver height were tested. The emitters were deployed at heights of 0.5 m, 1 m, 1.5 m, and 2 m. The receivers were placed at 0 m for the on-ground deployment, 0.5 m for the near-ground deployment, and 1.5 m for the above-ground deployment. The emitter and receiver were separated for each test. The trees are spaced in four-meter intervals and the field is located in an area with a Mediterranean climate. The foliage of the trees affects the wireless communication among the devices. Testing different configurations of transmitter and receiver provides us with the knowledge to design the best deployment for optimal communication with this type of crop. The tests were performed with sunny weather and temperatures of 20 °C. The measurement carried out is the received signal strength indicator (RSSI) at different measuring points. The Esp32 DevKit nodes were encapsulated on a protective box.

**Figure 14:**
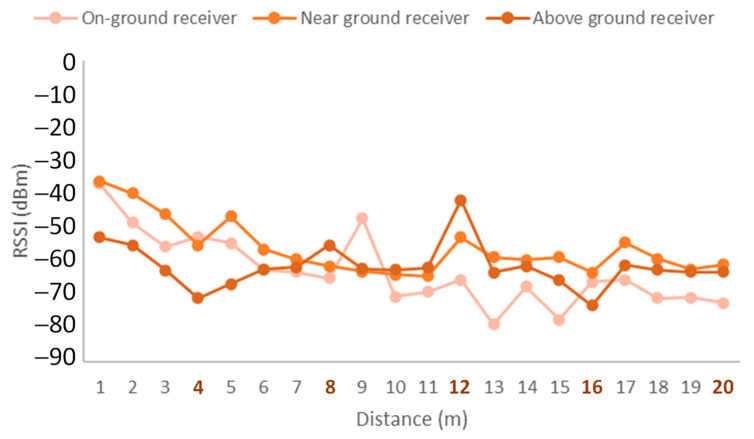
Emitter at height of 0.5 m.

The results for the emitter at a height of 0.5 m and the receiver at different deployment configurations are presented in Figure 14. The positions of the trees are indicated by the bold orange numbers on the X-axis. As can be seen, the overall higher RSSI values considering multiple trees along the tested distance were obtained for the near-ground position of the receiver. This configuration of the receiver is also the most stable. Moreover, some small fluctuations occurred for tree number 1 and tree number 4. However, the foliage in the space between trees 2 to 4 presented a higher density, which lead to higher fluctuations. One of the reasons for these fluctuations may be the multipath effect. Thus, avoiding node deployments in areas of high foliage density is best to obtain more stable signals.

**Figure 15:**
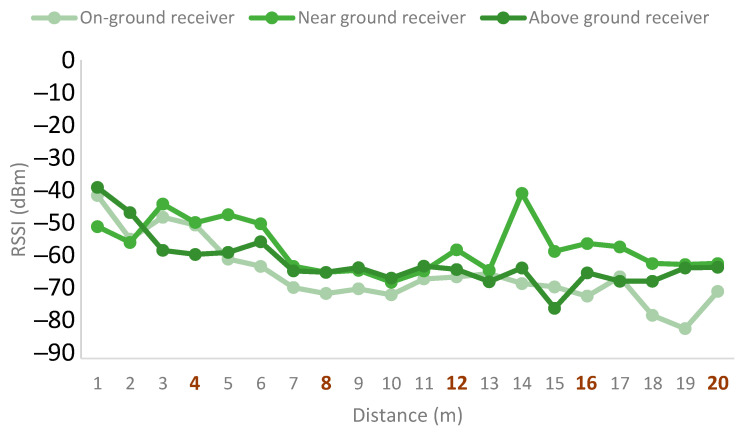
Emitter at height of 1 m.

For the case of the emitter deployed at a height of 1 m, the results are presented in Figure 15. As it can be seen, the near-ground receiver is the one with the best results. In this case, the signal quality is reduced between trees 2 and 4. However, for the near-ground receiver, the signal presents some recovery after the area with high foliage density. The above-ground deployment presents similar results for the area with high foliage density but worse signal quality for the rest of the measurement points. Lastly, the on-ground receiver deployment presents the worst results.

**Figure 16:**
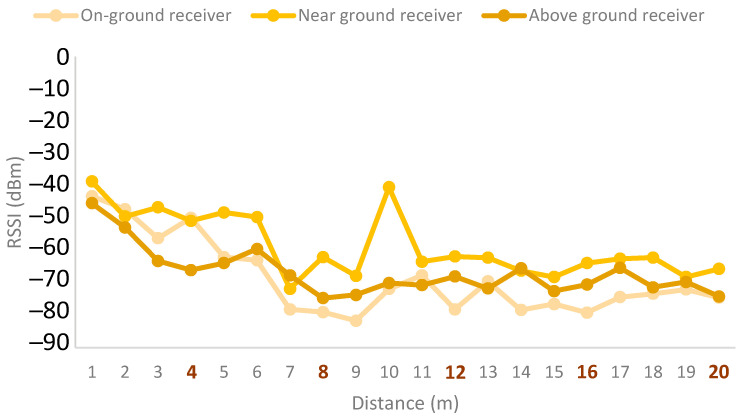
Emitter at height of 1.5 m.

Figure 16 presents the results for the emitter height of 1.5 m. The near-ground deployment has the highest signal quality values at almost all measuring points. As can be seen, it experiences some fluctuations between trees 2 and 3. However, even with the fluctuations, the signal quality is better than that of the other configurations. The next best option is the above-ground receiver. In this case, the signal is more stable while remaining below the quality levels of the near-ground receiver. Lastly, the on-ground deployment presented the worst results and the highest fluctuations. Another final aspect to consider is that the average signal quality for this emitter height was lower than the signal quality obtained for lower emitter heights. 

**Figure 17:**
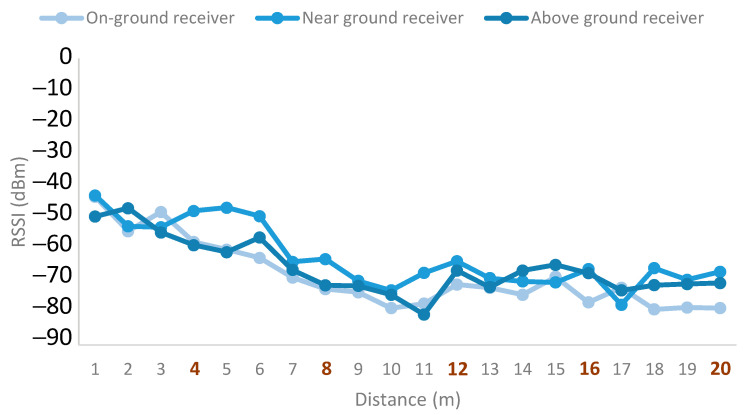
Emitter at height of 2 m.

Lastly, the results for the emitter height of 2 m are presented in Figure 17. This emitter height obtains the worst signal quality results compared to all the emitter heights. Regarding the receiver height, in this case as well, the near-ground deployment obtained the best results. However, as shown in the figure, all receiver configurations present similar results, while the results for this emitter height present the least fluctuations. As in the other cases, the on-ground configuration was the worst option.

Considering the results for all the emitter heights, we can conclude that in the case of orange groves, emitter heights of 0.5 and 1 m present the best signal quality and the near-ground receiver deployment is the best option for all emitter heights. Therefore, near-ground configurations are the optimal deployment style for both emitters and receivers.

The coverage results obtained from the tests performed on the orange groves have been utilized to obtain a heuristic signal attenuation model for all emitter heights as specified in [7]. The outlier values were discarded to perform this model. Equations (6)–(9) show the model for emitter heights of 0.5 m, 1 m, 1.5 m, and 2 m respectively.

(6) $$P_{0,5\;m}=-7.182\ln d\left(m\right)-45.276$$

(7) $$P_{1\;m}=-7.69\ln d\left(m\right)-44.194$$

(8) $$P_{1,5\;m}=-9.545\ln d\left(m\right)-44.475$$

(9) $$P_{2\;m}=-10.34\ln d\left(m\right)-43.493$$

**Figure 18:**
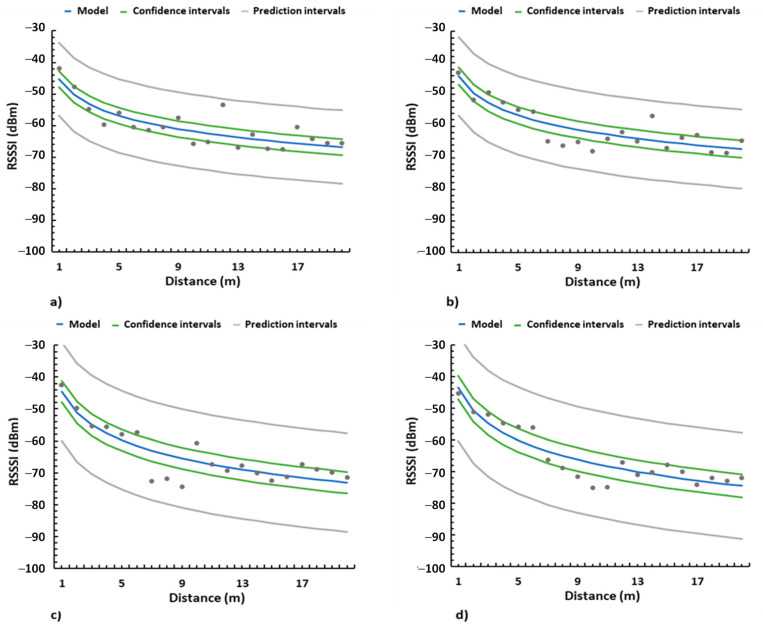
Heuristic model for (**a**) emitter at height of 0.5 m, (**b**) emitter at height of 1 m, (**c**) emitter at height of 1.5 m, and (**d**) emitter at height of 2 m.

Furthermore, the model, confidence intervals, and prediction intervals are presented in Figure 15, where the dots represent the values obtained from the tests on the fields. As can be seen, the model reflects that the configurations of emitter heights of 0.5 m and 1 m (See Figure 18a,b) present better signal quality. Lastly, Figure 18c shows the graphic representation for the case of the emitter height at 1.5 m and Figure 18d presents the results for the emitter height of 2 m.

Considering the results for all the emitter heights, we can conclude that for the case of orange groves, emitter heights of 0.5 and 1 m present the best signal quality and the near-ground receiver deployment was the best option for all emitter heights. Therefore, near-ground configurations are the optimal deployment style for both emitters and receivers.

## 6. Conclusions and Future Work

Estimating the amount of water needed to irrigate a crop is essential to carry out efficient use of a scarce resources such as water. The introduction of technology in the agricultural sector is also important to improve the sustainability and competitiveness of the sector. For this reason, this paper has presented the prototype of a low-cost sensor based on coils for measuring soil moisture. For this, three prototypes composed of two coils with different characteristics have been presented. These coils have been tested to analyze their behavior based on the humidity level of the soil. After the observed results, it has been concluded that the sensor that has had the best performance is prototype 1 working at 93 kHz. Additionally, a power circuit based on the ICM7555 has been designed to be able to generate the biphase signal to power the soil moisture sensor. This sensor is able to measure the percentage of water content in the soil at the desired depth. This fact helps us to ensure the correct irrigation of the root ball. The sensor and power supply circuit is connected to an ESP32 module for reading and storing humidity measurements. The entire system has been tested with real samples for the extraction of its mathematical behavior model. The results show that our sensor demonstrates that by using these models we can achieve accuracies close to 95%. 

Additionally, the network performance has been tested in a real, cultivated plot. According to the results, and after modeling mathematically the results of the network coverage, we can conclude that for the case of orange groves, the best results are obtained when the emitter is placed at 0.5 and 1 m and the receiver is placed near the ground. So, near-ground configurations are the optimal deployment style for both emitters and receivers.

In future work, we would like to perform more practical experiments with more models of coils and different kinds of soils to design a more versatile sensor capable of working with several sorts of soils without changing the sensor. It will also study the possibility of including a system to automatically adapt the working frequency to the type of soil. Because in our practical experiments we have included only the measurements of signal amplitude, it could be interesting to measure the quadrature component and phase of the obtained signal and trying to relate these parameters with changes of pH of water. We also want to include other sensors in a multi-parametric node to place in the crop field [55,56] to enhance the efficiency of water management in precision agriculture [57]. In this sense, we want to check if soil temperature has some effect over the soil moisture measurements and, if required, over obtaining the soil moisture values compensated with temperature. Finally, as the last step, we will study the most appropriate enclosures to protect our entire system. 

## Figures and Tables

**Table 1:** Previous studies regarding the use of WSNs in soil monitoring for agriculture.

Types	References
Surveys of WSN Implementations for Agriculture.	[1,8,9,10,11,12,13,14]
Frameworks, Studies, Designs and Deployments for WSN.	[7,15,16,17,18]
WSNs for the Monitoring of Specific Crops.	[19,20]
WSNs in Greenhouses.	[21,22]
Energy Savings Studies.	[23,24]

**Table 2:** Prototypes to measure soil moisture.

Prototype	Caliber	N° Layers	N° Spires 1st Coil	N° Spires 2nd Coil	N:n	Diameter
P1 (Figure 4a)	0.6 mm	1	15	30	1:2	50 mm
P2 (Figure 4b)	0.6 mm	1	15	30	1:2	32 mm
P3 (Figure 4c)	0.6 mm	1	15	30	1:2	20 mm

**Table 3:** Samples used during the tests.

Sample	Mass of Dry Soil (g)	Mass of Wet Soil (g)	Mass of Water (g)	% Volumetric Water Content
1	4000	4000	0	0
2	4000	4250	250	6.25
3	4000	4500	500	12.5
4	4000	4750	750	18.75
5	4000	5000	1000	25

**Table 4:** Prototypes to measure soil moisture.

Prototype	Working Frequency (kHz)	Maximum Voltage (mV)
1	93	1820
2	146	1570
3	216	680

**Table 5:** Comparison of soil moisture sensors with our proposal.

Ref.	Model	Sensitivity	Power	Size	Cost
[42]	RK520-02 Soil Moisture Sensor, Temperature Probe & EC Sensor	0–100%	5 VDC, 12–24 VDC	136 × 45 mm	$58.00–$72.00
[43]	S-Soil MT-02A	±3% (0–53%) ±5% (53–100%)	3.6–30 VDC	149 × 45 mm	$79.00
[44]	S-Temp&VWC&EC-02A	±2% (0–50%) ±3% (50–100%)	3.6–30 VDC	149 × 45 mm	$99.00
[45]	SenseCAP Wireless Soil Moisture & Temperature Sensor	±2% (0–50%); ±3% (50–100%)	3.6 V	149 × 45 mm	$219.00
[46]	Sensor YL-69	0–95%	3.3–5 VCD	60 × 30 mm	$2.65
[47]	Keyes Brick Soil Moisture Sensor Module	-	3.3–5V VDC	63 × 22 × 8 mm	$1.34
[48]	KeeYees	-	3.3–5 VDC	3.858 × 0.906 mm	$7.99
[49]	Seeed Studio Grove—Capacitive Moisture Sensor—101020614	-	3.3–5 V	-	$5.95
[50]	Grove—Soil Moisture Sensor	0–95%	3.3–5 V	60 × 20 × 6.35 mm	$2.99
[51]	Seeed Studio Moisture Sensor—101020008	-	3.3 V CC y 5 VDC	-	$3.99
[52]	SEN0308	0–57%	3.3–5.5 VDC	175 × 30 mm	$15.51
[52]	MSE020SMS	-	3.3V–12 VDC	-	$5.80
-	Our proposal	-	9 V	50 m × 30 m	$2.15

## Data Availability

No dataset has been created.
